# Experimental and Theoretical Investigation of the Inhibitor Efficiency of *Eucalyptus globulus* Leaf Essential Oil (*EuEO*) on Mild Steel Corrosion in a Molar Hydrochloric Acid Medium

**DOI:** 10.3390/molecules29143323

**Published:** 2024-07-15

**Authors:** Dounia Azzouni, Soukaina Alaoui Mrani, Roberta Bertani, Mohammed M. Alanazi, Ghizlan En-nabety, Mustapha Taleb

**Affiliations:** 1 Laboratory of Engineering, Electrochemistry, Modelling and Environment, Faculty of Sciences, Sidi Mohamed Ben Abdellah University, Fez 30000, Morocco; soukaina.alaouimrani@usmba.ac.ma (S.A.M.); nabetyghizlan@gmail.com (G.E.-n.); mustapha.taleb@usmba.ac.ma (M.T.); 2 Industrial Engineering Department, University of Padua, Via F. Marzolo 9, 35131 Padua, Italy; roberta.bertani@unipd.it; 3 Department of Pharmaceutical Chemistry, College of Pharmacy, King Saud University, Riyadh 11451, Saudi Arabia; mmalanazi@ksu.edu.sa

**Keywords:** *EuEO*, characterization, corrosion inhibition, electrochemical study, theoretical study

## Abstract

As a corrosion inhibitor for mild steel in a molar hydrochloric acid medium, we investigated the potential of *Eucalyptus globulus* essential oil (*EuEO*). Through electrochemical impedance spectroscopy (EIS), potentiodynamic polarization curves, and theoretical methods, including DFT/B3LYP 6-31G (d, p) and Monte Carlo simulations, the interactions between the *EuEO* components and the steel surface were analyzed. D-Allose, Betulinaldehyde, and Uvaol were identified as the major active compounds in the GC-MS analysis. The experimental results showed that *EuEO* reached an inhibitory efficiency as high as 97% at a 1 g/L concentration. The findings suggest that *EuEO* operates as a mixed-type inhibitor, reducing both cathodic and anodic reactions, as well as building up a protective coating on the steel surface. Simulations also confirmed that *EuEO* molecules function as electron donors and acceptors, enhancing corrosion resistance.

## 1. Introduction

Steels are iron-based alloys that are widely used in industry because of their good mechanical and physicochemical properties and low cost. These qualities enable them to be used in a variety of industries, including food packaging, electronics, building and construction, and the petroleum sector [1]. Corrosion phenomena have been documented for as long as humans have managed to prepare metals that are not naturally occurring in a pure state. They are the outcome of an environment’s chemical or electrochemical reaction with metals and alloys [2]. It is also evident that, aside from metals like platinum or gold, which are found in the soil as metals, corrosion only returns metals to their original states as oxides, sulfides, or carbonates. Metal structures deteriorate quickly as a result of these processes [3]. As a result of having to repair broken parts, sectors suffer huge economic losses. It is challenging to assess both the direct and indirect costs related to corrosion’s effects. These specifically include lost production time for some businesses and higher maintenance expenses due to damaged infrastructure [4]. The hydrodynamic regime applied to the material, the stresses placed on it, the nature and structure of the material, surface treatments (mechanical, chemical, electrochemical, etc.), the environment and its chemical properties, temperature, and many more factors are involved in the corrosion process. Metallic materials are especially vulnerable to degradation in any hostile environment [5]. These days, anticorrosion protection involves a wide range of procedures, including passivation, sacrificial anode protection, cathodic and anodic surface treatments, and inhibitor-based environmental intervention. In actuality, inhibitors are a novel approach to stopping corrosion [6]. Although most corrosion inhibitors are dangerous for both humans and the environment, they are most frequently used to increase the durability of metals, particularly in acidic media [7]. Because of their toxicity, natural substances have been selected for corrosion inhibitor research due to their effectiveness, environmental friendliness, and lower cost than synthetic solutions. They are renewable, biodegradable, and readily available [8]. Essential oils have garnered interest for their anticorrosive action in recent years. It is common practice to assess the inhibitory power of essential oils in the presence of hydrochloric acid as a corrosive solution [9,10,11,12,13,14,15].

In a molar hydrochloric acid medium, this study aims to examine the potential of the essential oil from *Eucalyptus globulus* (*EuEO*) leaves as a corrosion inhibitor for mild steel. Numerous experimental techniques, including electrochemical impedance spectroscopy (EIS) and potentiodynamic polarization curves, were used to this end. We further supplemented our analysis with a theoretical approach with the basis set DFT/B_3_LYP 6-31G (d, p). In order to gain a better understanding of the interactions between the primary oil components and the steel surface in an acidic medium, a Monte Carlo simulation was also conducted.

## 2. Result and Discussion

### 2.1. Characterization of Eu and EuEO

**Figure 1:**
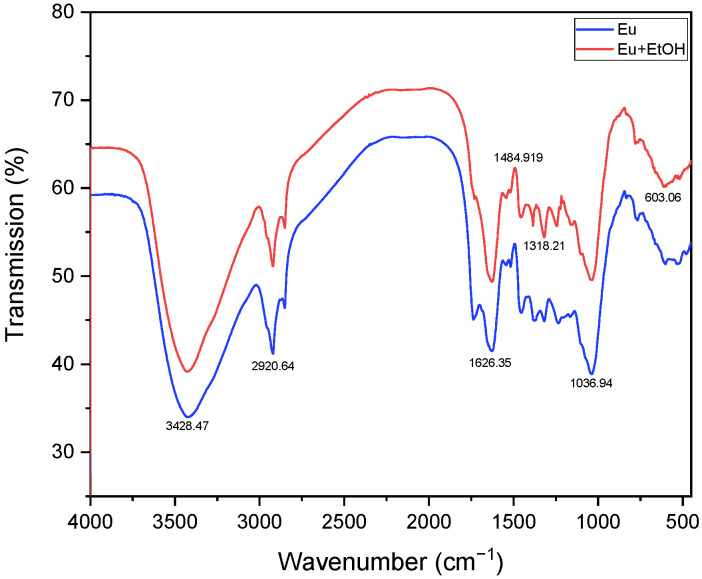
FTIR spectra of Eu and *EuEO*.

The FTIR technique was used to identify the various functional groups of Eu and *EuEO*. Figure 1 shows the FTIR spectra of Eu and *EuEO*. A strong peak is seen at 3428 cm^−1^, which is associated with the absorbed O-H stretching vibrations of cellulose and water molecules [16,17]. The C-H stretching vibrations of the methyl and methylene groups in cellulose are responsible for the peaks that occur between 2900 and 3000 cm^−1^ [18]. Peaks ranging from 1300 to 1800 cm^−1^ might be associated with phenolic functional groups, like aromatic ring vibrations and C=O stretching vibrations [19]. The presence of acetyl groups in hemicellulose may be indicated by additional peaks at about 1740 cm^−1^ [20]. Indicative of aromatic C=C stretching vibrations in lignin is the peak at 1626 cm^−1^. Peaks in the 1200–1500 cm^−1^ range are linked to several functional groups in lignin, including vibrations that stretch and bend the C-O and C-H bonds [21]. C-O-C and C-O stretching vibrations in hemicellulose are characterized by peaks in the range of 900–1200 cm^−1^ [22,23]. We saw some peaks in the *EuEO* spectrum reduce, which indicated that the eucalyptus leaf oil had been extracted.

**Figure 2:**
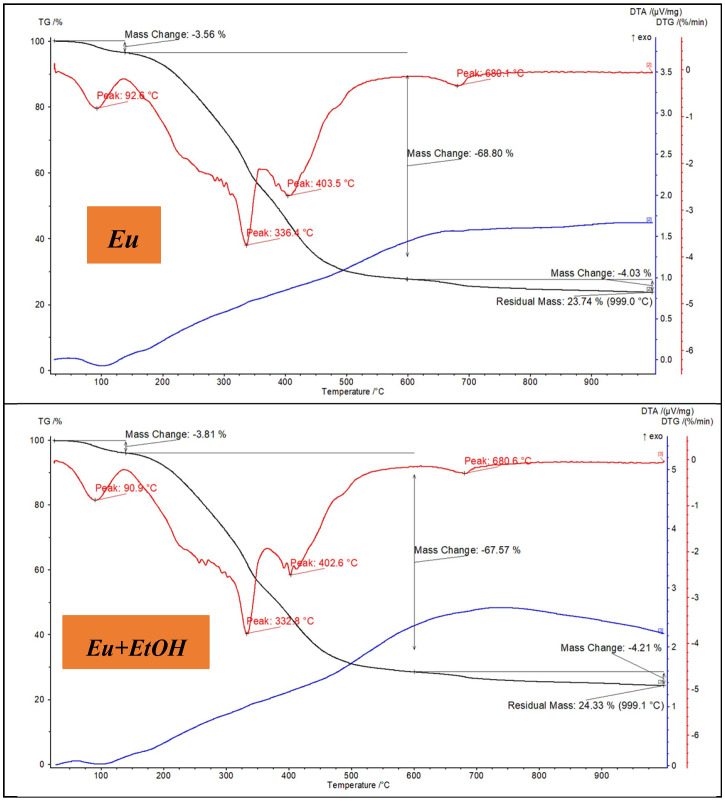
TGA/TDA curves of Eu and *EuEO*.

Regarding its moisture content, breakdown profile, and thermal stability, TGA/DTA can offer important details. Figure 2 displays the thermal analysis of Eu and *EuEO*. The sample’s moisture content evaporating may cause a slight initial weight loss. At low temperatures (<200 °C), this can be seen as a downward trend in the TGA curve [24]. Different temperatures are required for the breakdown of cellulose, hemicellulose, and lignin, which are the primary constituents of Eu and *EuEO* [25]. The thermal stability and breakdown kinetics of these components can be understood using TGA/DTA [26]. Generally, cellulose breaks down between 300 and 400 °C, hemicellulose between 200 and 300 °C, and lignin between 250 and 500 °C [27]. Higher temperatures may cause additional pyrolysis or oxidation of the char residue left over from the breakdown of Eu and *EuEO* [28]. Whereas DTA can identify any exothermic or endothermic events connected to char generation or combustion, TGA can measure the quantity of char residue that remains after decomposition.

**Figure 3:**
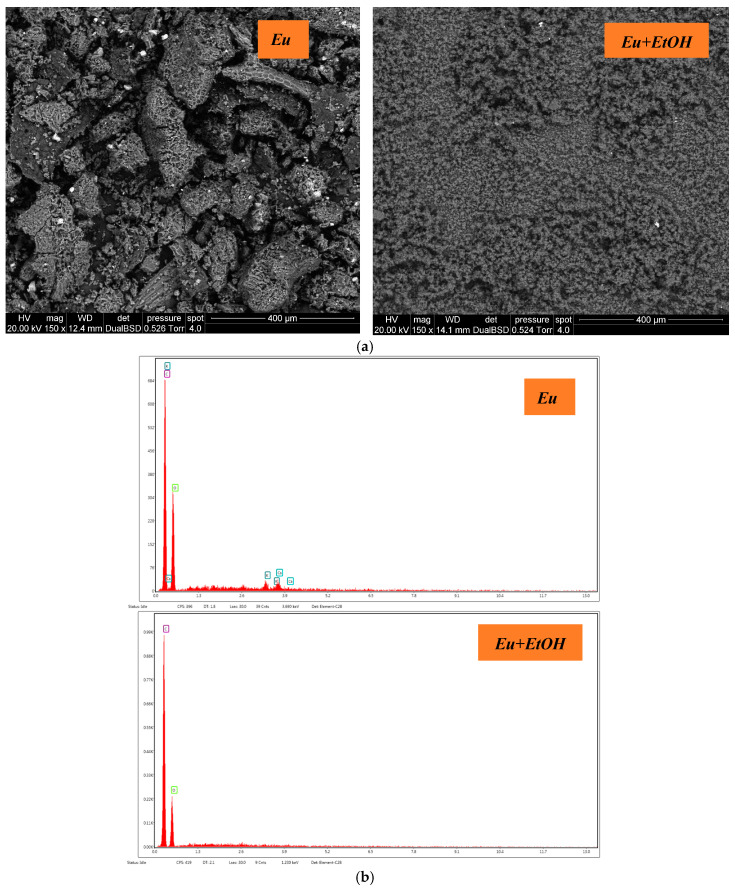
ESEM images (**a**) and EDX elementals (**b**) of Eu and *EuEO*.

Figure 3a,b displays the EDX elementals and ESEM pictures for Eu and *EuEO*. The microstructure of the oil glands in eucalyptus leaves which produce and store essential oils can be better understood using ESEM imaging. On the surface of the leaves, oil glands can be seen as glandular trichomes or spherical formations. Qualitative data regarding Eu and *EuEO*’s elemental makeup can be obtained using EDX analysis. While EDX analysis confirms that the spatial distribution in Eu is consistent with C (49.66%), O (46.5%), K (1.63%) and Ca (2.21%), and EuEO exhibits different percentages of C (64.53%) and O (35.47%), the elements K and Ca are absent (Table 1).

### 2.2. Essential Oil Composition

The essential oil’s composition may differ according to greenhouse type, covering material, and country of origin. As reported in the literature, chromatographic analysis and mass spectroscopy of *Eucalyptus globulus* essential oil reveal that it contains mainly monoterpenes (hydrocarbons and oxygenated compounds), such as eucalyptol, linalool, α-pinene, β-pinene, β-myrcene, α-terpinene, p-cymene, ocimene, terpinen-4-ol, α-terpineol, and geraniol. Oxygenated sesquiterpenes and sesquiterpenes such as spathulenol, globulol, β-selinene, β-eudesmol, δ-cadinene, and γ-elemene were also identified [29,30,31]. However, in the present study, the analysis of *EuEO* using the GC-MS technique facilitated the identification of several components. The chemical structures of the three most abundant major constituents in *EuEO* are presented in Table 2.

### 2.3. Electrochemical Study

The mechanisms behind the corrosion process cannot be understood by measuring anticorrosive performance as a function of weight. However, we need to apply electrochemical techniques to explain physical properties like the double-layer capacity, transfer resistance, and film capacity at the metal/solution interface.

In multiple trials at varying doses, the inhibitory effectiveness of *EuEO* against mild steel corrosion in 1 M HCl solution was assessed.

#### 2.3.1. Open Circuit Potential (OCP) Monitoring

The electrode needs to be submerged in the electrolyte for a period of time equal to the solution’s electrochemical equilibrium before beginning any electrochemical test [32,33].

In this work, we propose that a 100% iron metal plate submerged in a molar hydrochloric acid solution be stabilized by immersion for 30 min. This will offer some initial insights into the reactions taking place at the interface between the metal and electrolyte.

**Figure 4:**
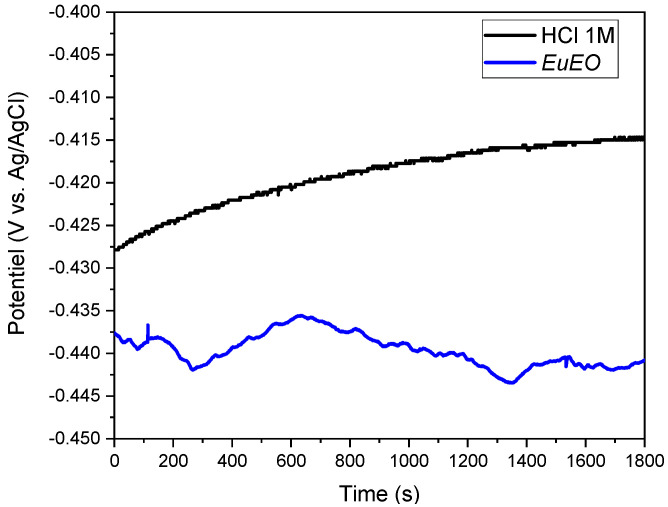
Open circuit potential evolution of mild steel in 1 M HCl solution with and without the inhibitor at 298 K.

Figure 4 shows the OCP variations with time for the mild steel electrode in a corrosive solution at 298 K with and without 1 g/L of *EuEO*. The principal effect of these chemicals on the cathodic process was demonstrated by the potential values shifting towards greater negative values. It also seems that equilibrium potential can be reached in thirty minutes or less.

#### 2.3.2. Polarization Curves

**Figure 5:**
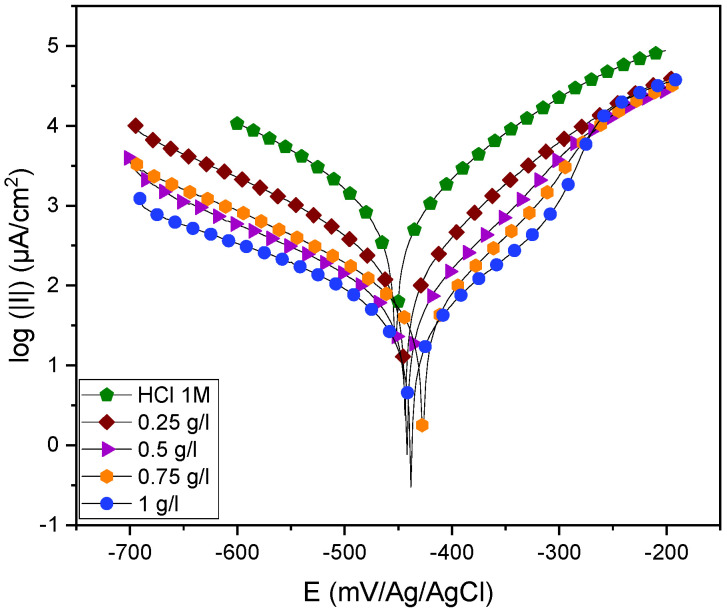
Mild steel polarization curves in 1 M HCl medium without and with the addition of different concentrations of *EuEO*.

Figure 5 displays the polarization curves obtained after 30 min of stabilization, both with and without the inhibitor under study. In order to prevent the iron below from contaminating the electrolyte, the potential was swept to the maximum cathodic potential.

The cathodic branches of the polarization curve were clearly trending parallel after *EuEO*’s addition into the HCl solution, indicating that *EuEO* had no effect on the reactive mechanism of cathodic hydrogen evolution. The slope of the anodic branch curve was significantly modified, indicating that after *EuEO*’s adhesion to the steel/acid interface, a compact sequential barrier film formed on the steel/acid interface, thus retarding steel/acid oxidation of the anodic Fe atoms [32,33]. Furthermore, at a −300 mV Ag/AgCl polarization potential, a platform inflection point appears at the anodic branch, which indicates that *EuEO’s* adhesion to the mild steel interface reaches a state of saturation at this polarization potential and starts to produce a desorption phenomenon to a limited extent [33,34,35]. Table 3 lists the corrosion current density (i_corr_), corrosion potential (E_corr_), cathodic and anodic Tafel slope (β_c_ and β_a_), and inhibitory efficiency (IE_pdp_ %) at various concentrations of the inhibitor tested. These parameters were extracted from the curves using EC-LAB V11.27 software.

The cathodic Tafel slope (β_c_) experiences a slight alteration when the inhibitor is added into an acidic medium. The inhibitor addition only affects slightly their proton reduction pathway, which is consistent with the Heyrovsky model, as demonstrated by the β_c_ values. This permits the studied inhibitor to simultaneously cover as many cathodic sites as anodic sites [33,36,37].

Table 3’s corrosion current densities show a decrease with an increasing inhibitor concentration, which subsequently results in a decrease in the anodic and cathodic reactions in the absence of the inhibitor. This suggests that the dissolution of mild steel at the anode and the cathodic reduction of hydrogen ions have been permanently inhibited, which lowers the rate of electrochemical reaction by forming a protective layer on the mild steel’s surface.

It is also important to note that the inhibition efficiency rises with the inhibitor concentration, reaching a higher value of 1 g/L, or 97%, demonstrating the great efficacy of *EuEO* as an inhibitor of steel corrosion in 1 M HCl solution. We now have a comprehensive understanding of the steel dissolution kinetics in a corrosive liquid thanks to this stationary approach. On the other hand, the sequence of the fundamental stages of the mechanisms occurring at the metal/solution contact cannot be determined without electrochemical measurements utilizing a transient approach [33,38,39].

#### 2.3.3. Electrochemical Impedance Spectroscopy Measurements

It was necessary to use electrochemical impedance spectroscopy (EIS) to validate the polarization method’s results. This method is frequently employed to assess the protective film’s dielectric qualities and clarify the inhibition mechanism. Furthermore, EIS makes it easier to comprehend the chemical or electrochemical reactions taking on inside the resultant layers [33,40,41].

#### 2.3.4. Mild Steel/1 M HCl Interface Equivalent Electrical Circuits

**Figure 6:**
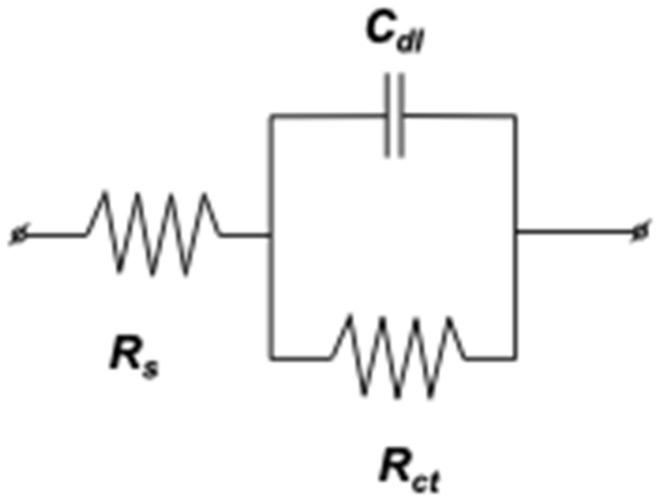
Proposed equivalent electrical circuit model (CPE) for modeling the behavior of mild steel in a molar HCl environment.

The purpose of equivalent electrical circuits (CPE) is to determine the critical variables needed to comprehend the corrosion process as a whole [42]. Thus, using a single-time-constant electrical circuit of the modified Randles R_s_ + CP_Edc_/R_p_ type, impedance diagrams corresponding to mild steel in the presence and absence of various *EuEO* concentrations in a molar hydrochloric acid medium are modeled on the Nyquist and Bode planes (Figure 6).

#### 2.3.5. Representation of Impedance Diagrams on the Nyquist and Bode Planes

**Figure 7:**
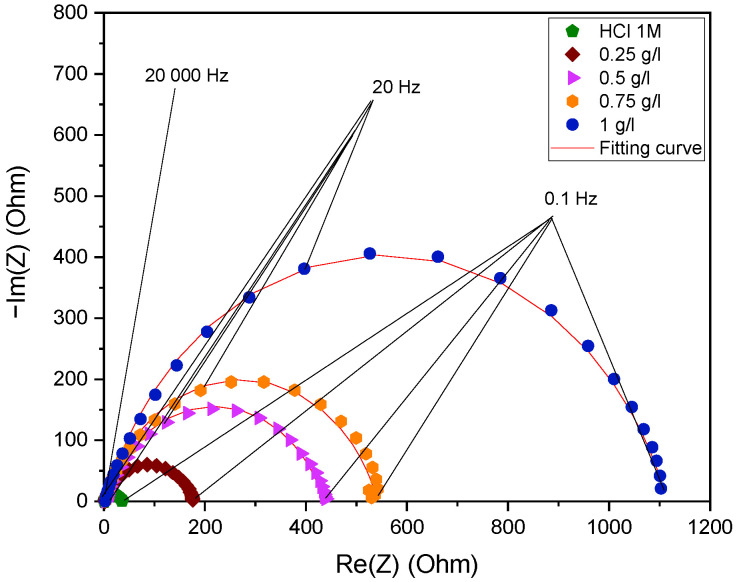
Electrochemical impedance plots on the Nyquist plane in the presence and absence of the studied inhibitor at corrosion potential in 1 M HCl solution.

The impedance graphs on the Nyquist plane following 30 min of immersion in a molar hydrochloric acid medium with *EuEO* are shown in Figure 7. The electrochemical properties of the metal/solution interface, including the resistances (R_s_ and R_p_) and values (Q and n_dl_), are obtained directly from the fitting impedance diagrams by matching the impedance diagrams with the corresponding electrical circuits [33].

**Figure 8:**
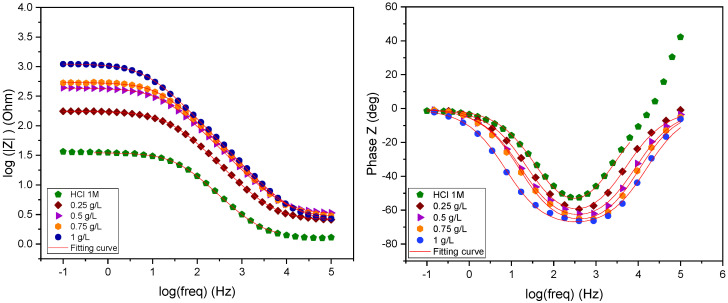
Electrochemical interface Bode plots without and with inhibitor at different concentrations.

In order to track variations in the phase shift (φ) in relation to the frequency logarithm (Log (f)) and the logarithm of the impedance modulus |Z|, impedance diagrams are also shown on the Bode plane. This gives insight into the mechanisms that operate at higher frequencies and that the Nyquist representation may not always be able to accurately depict. Bode plane impedance diagrams for mild steel are shown in Figure 8 with and without different concentrations of *EuEO* in 1 M HCl [33].

During inhibition, the impedance diagrams take the shape of asymmetric semicircles that are centered on the reference axis. However, because of the metal surface’s general stiffness and heterogeneity, there may be a relationship between this and the frequency dispersion within the interracial impedance [33,43,44]. When *EuEO* is included, the mild steel solution mechanism in the medium under investigation does not change, as evidenced by the constant shape of the Nyquist diagrams [33,45,46]. Additionally, these diagrams grow larger with concentration, providing better corrosion resistance [33,47].

Due to the surface heterogeneity, the impedance spectra obtained in 1 M HCl media for steel in the presence of *EuEO* cannot be reconciled with traditional constant phase element (CPE) analysis. To address the issue of semicircle flatness associated with surface inhomogeneity, constant phase elements (CPE) were used in place of pure capacitance. With the modified Randles model, this replacement made it possible to obtain an excellent parametric fit of the observed impedance spectra on the Nyquist plane. This emphasizes how well constant phase element (CPE) work with frequency distribution.

Table 4 summarizes the data derived from the interface modeling. It shows that in the absence of the inhibitor, the depolarization resistance (R_p_) is around 33.97 Ω.cm^2^, but in the presence of the inhibitor, it rises to 1113 Ω.cm^2^ at a dosage of 1 g/L. When inhibitors are present, they adsorb on the metal surface and form a protective coating that stops reactions on the iron electrode’s surface, which causes depolarization resistance [33,48]. Additionally, it is noted that at a concentration of 1 g/L of inhibitor, the pseudo-capacity of the double layer (C_dl_) drops from 117 μF.Cm^−2^ in corrosive solution to 19.69 μF.Cm^−2^. The development of a protective layer on the steel surface is the cause of this reduction. The electrode is replaced by water molecules and other ions that are first adsorbed by the inhibitor molecules, creating a protective coating, which is the cause of the electrode capacity loss. The Helmholtz theory, however, states that if the inhibitor is adsorbed, the organic film’s density rises and the double layer’s capacity falls [33,49,50,51]. Consequently, the Q values fall and are lower than those tested without an inhibitor as the inhibitor concentration increases noticeably. This implies that this metric is greatly impacted by the inhibitor’s concentration. For the inhibitor under study, this pattern is also seen for the other film characteristics (film capacitance and resistance). Increasing the inhibitor concentration is also correlated with an increase in inhibitory efficacy. It should be noted that the difference in the molecular structure is responsible for the maximal inhibitory efficacy of around 97% at 1 g/L. Multiple sites enhance interaction with the iron surface, *EuEO* is thus adsorbed more onto the mild steel surface. Figure 8 shows a Bode diagram that plots the interfaces obtained with 1 M HCI solution and in the presence of various doses of the inhibitor under study. The frequency-dependent behavior exhibits a minimum at intermediate frequencies, as these figures clearly demonstrate [33,52,53], suggesting that the time constant of the pure charge process is uniform. Additionally, as the inhibitor concentration rises in the corrosive 1 M HCl medium, the Bode plots demonstrate an increase in both the Bode modulus and the phase slope. These findings demonstrate that the adsorption activity of the investigated inhibitor causes the metal/solution interface to exhibit capacitive behavior at the mild steel electrode’s surface [33,54].

### 2.4. Adsorption Isotherms

It is crucial to recognize the type of adsorption isotherm, chemisorption, physisorption, or mixed, to understand the process of organo-electrochemical reactions [55].

**Figure 9:**
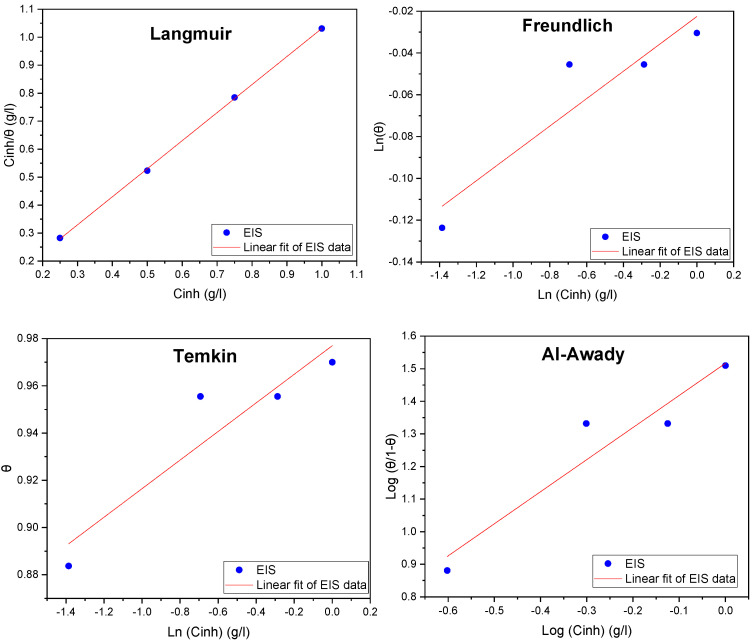
Adsorption isotherm plots of *EuEO*.

As illustrated in Figure 9, the surface coverage obtained from the impedance measurements is examined using the Langmuir, Temkin, Freundlich, and El-Awady isotherm models. Equations (1)–(4) provide the adsorption isotherms that relate surface coverage (θ) and inhibitor concentration (C_inh_), as follows [56]:

(1) $$\mathrm{Langmuir}:\frac{C_{inh}}{\theta }=\frac{1}{K_{ads}}+C_{inh}$$

(2) $$\mathrm{Freundlich}:Log(\theta )=Log(K_{ads})+ZLog(C_{inh})$$

(3) $$\mathrm{Temkin}:\theta =\frac{-1}{2a}\ln(K_{ads})+\frac{-1}{2a}\ln(C_{inh})$$

(4) $$\mathrm{El}\text{-}\mathrm{Awady}:Log(\frac{\theta }{1-\theta })=yLog(K_{ads})+yLog(C_{inh})$$

where K_ads_ is the equilibrium constant of the adsorption/desorption process, C_inh_ is the inhibitor concentration, and θ is the inhibitor surface coverage.

Various adsorption isotherm models using the EIS technique are represented in Figure 9. Additionally, the isotherms and their parameters are listed in Table 5.

The results in Table 5 make it evident that the regression coefficient R^2^ fits all four isotherms perfectly. Even so, the Langmuir isotherm’s slope is very near to unity and remains the most accurate way to describe the adsorption process. This implies that an alternative isotherm that more accurately captures the corrosion process needs to be found [13,14]. The El-Awady isotherm value 1/y is close to 1, indicating that during the inhibition process, each active component in the essential oil is replaced by a water molecule bound to the active site on the mild steel surface [13,14].

The coefficient of determination R^2^ for all four isotherms is shown to be close to unity, and the K_ads_ value is highest for the Temkin isotherm. Consequently, the other models are rejected. We can thus verify that the adsorption process adheres to the Temkin isotherm given the high value of K_ads_ and the R^2^ coefficient. Additionally, the presence of molecular interactions in the adsorbed layer is suggested by the fact that the intermolecular interaction parameter between the essential oil molecules adsorbed onto the mild steel surface reveals a negative value [13,14].

In addition, the adsorption standard free energy, ΔG^0^_ads_, was calculated according to Equation (5):

ΔG^0^_ads_ = −RT ln (1000 × K_ads_) (5)

where R is the universal gas constant, T is the temperature equal to 298 K, K_ads_ is the equilibrium constant of the adsorption process, and 1000 g/L refers to the concentration of water in the solution.

The interactions between the charged essential oil components and the charged mild steel surface are usually electrostatic, and adsorption occurs physically, at least at −20 kJ.mol^−1^. Conversely, with negative values of −40 kJ.mol^−1^ or higher, the adsorption is chemical and involves electron transfer from the inhibitor molecules to the metal surface [57,58]. These findings indicate the possibility of chemisorption in *EuEO* essential oil adsorption.

### 2.5. Surface Analysis

**Figure 10:**
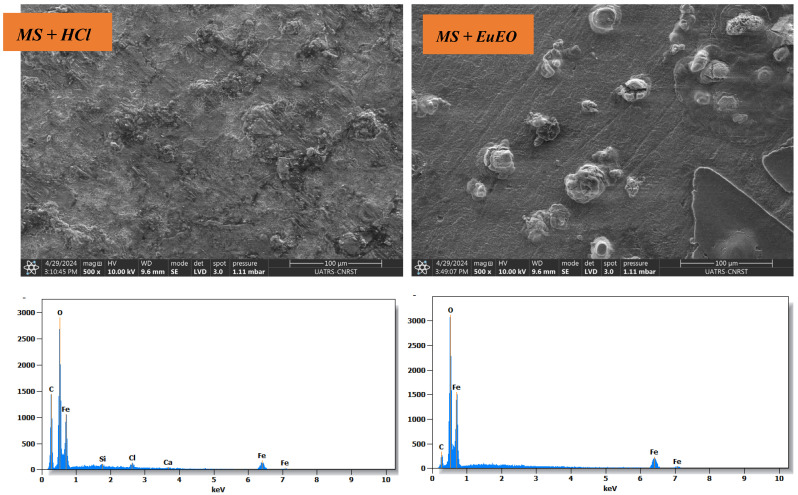
SEM/EDX image of mild steel surface after 6 h of immersion in 1 M HCl solution and after immersion in 1 M HCl solution with 1 g/L *EuEO*.

To better explain the adsorption process during corrosion inhibition, the surface morphology and EDX spectra were studied, and the results are shown in Figure 10.

Mild steel samples were immersed in 1 M HCl solution for 6 h, and energy-dispersive X-ray (EDX) analysis of the surface composition of the mild steel before and after protective film formation was performed with and without the optimal *EuEO* concentration. The electrochemical EDX spectrum for mild steel after immersion in 1 M HCl solution without the inhibitor shows the main components of the surface elements of mild steel; the chloride ion peak disappears in the presence of the inhibitor. By coating mild steel surfaces with these compounds, the atomic oxygen content was reduced from 28.9% to 25.3% (Table 6).

### 2.6. Evaluation of the Quantum Chemical Calculations

#### 2.6.1. DFT Calculations

DFT simulations were conducted at B_3_LYP/6-31G (d, p) for all three main components of *EuEO*, namely d-Allose, Betulinaldehyde, and Uvaol, in the aqueous phase to acquire additional insights into the essential oil’s interaction with the metal surface.

**Figure 11:**
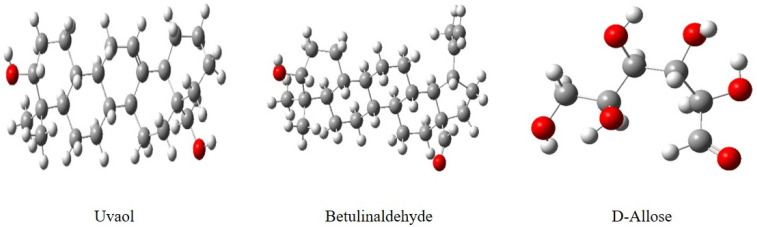
*EuEO* principal component optimized geometries, based on B_3_LYP/6-31G (d, p), in aqueous phase.

The findings of quantum computation make it abundantly evident that there are no imaginary frequencies in the vibrational spectra at all, indicating that the equilibrium structures represent the components with the lowest total energy *E_T_* values. Accordingly, as Figure 11 illustrates, the simulated structures that were obtained represent the optimum final geometries of these molecules.

DFT can be used to examine the electronic characteristics of an extensive spectrum of compounds, providing global descriptors of reactivity. Table 7 provides these properties for the compounds that were examined in the aqueous phase.

The energy of molecular orbitals can be used to determine a molecule’s overall reactivity. The most occupied (HOMO) and least occupied (LUMO) molecular orbitals of the reacting species interact to produce the electronic transition, according to the frontier molecular orbital (FMO) hypothesis of chemical reactivity. This describes how easily electrophiles can attack a molecule [59]. Strong values of E_HOMO_ can be used to predict a molecule’s tendency to donate electrons to other molecules with low-energy or vacant electronic orbitals that accept the appropriate electrons. On the other hand, LUMO energy describes a molecule’s vulnerability to nucleophile attack and is strongly correlated with electron affinity [60]. When it comes to molecules, the electron-accepting capacity increases with a decreasing LUMO value.

Table 7 illustrates that after adsorption onto the mild steel surface, electron donation and electron acceptance increase. According to the baseline structure, there is an increase in the E_HOMO_ function parameters and a decrease in the E_LUMO_ function values. An improved descriptor for describing a molecule’s overall reactivity is its energy gap (ΔE) [61,62]. The chemicals under investigation in this study have ΔE values in the following order: Uvaol < Betulinaldehyde < d-Allose. This indicates that d-Allose is the least reactive of the chemicals, and Uvaol and Betulinaldehyde are more reactive. Furthermore, the energy gap (ΔE) values exhibit a moderate increase in classification, indicating a higher degree of interaction related to the solvent’s polarity.

An organic molecule with a higher softness value and a lower hardness value is associated with a higher chemical reactivity, and vice versa. In this study, the chemical hardness η and chemical softness σ values of the three molecules can be classified into the following order: Uvaol (η = 2.6322) < Betulinaldehyde (η = 3.8083) < d-Allose (η = 4.0711) and Uvaol (σ = 0.3799) > Betulinaldehyde (σ = 0.2626) > d-Allose (σ = 0.2456), respectively. From these findings, both Uvaol and Betulinaldehyde are relatively more reactive. According to the literature, a ΔN value below 3.6 indicates that a molecule tends to donate electrons to metal surfaces. As Table 6 demonstrates, all the ΔN values calculated are below 3.6 [13,14].

**Figure 12:**
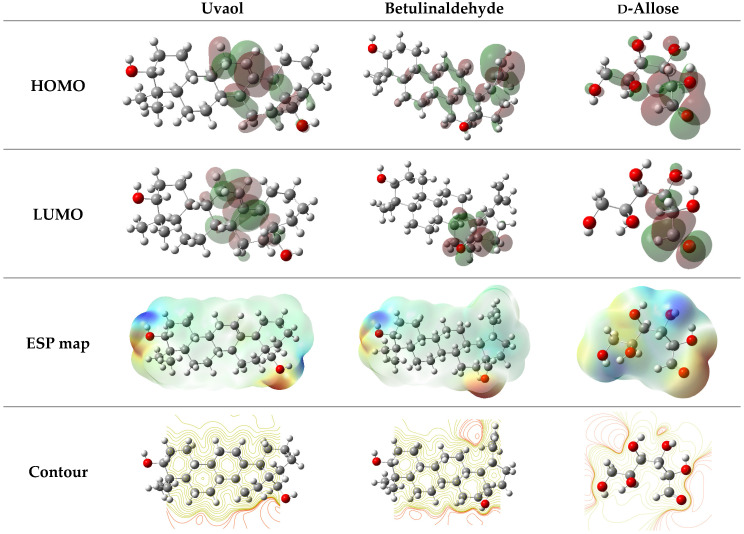
ESP maps, contours, and LUMO and HOMO density of Uvaol, Betulinaldehyde, and d-Allose.

Figure 12 illustrates the electronic density of the HOMO and LUMO atomic orbitals of the molecules under study, in addition to the geographic distribution of the electrostatic potentials (ESP map). The preferred active electron-donating HOMO sites are predominantly found in the red zone around the molecular heteroatoms (oxygen), as can be observed from the ESP map distributions [44]. Thus, it makes sense to speculate that heteroatoms provide electrons and have strong donor acceptor interactions with the unoccupied metal orbitals [63].

#### 2.6.2. Monte Carlo Simulation

**Figure 13:**
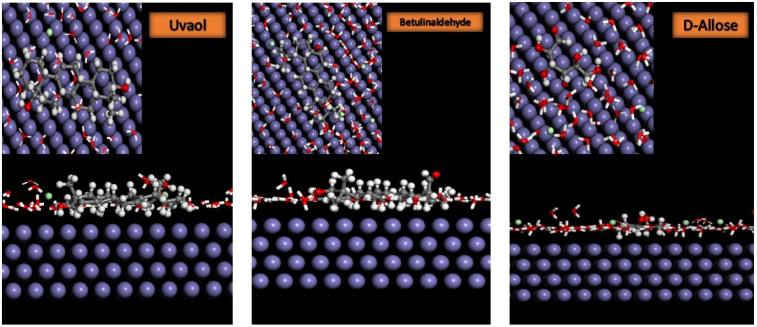
Top and side views of the most stable low-energy adsorption configurations of Uvaol, Betulinaldehyde, and d-Allose in Fe (110)/100 H_2_O system using the Monte Carlo simulation.

To better understand the adsorption behavior, dynamic Monte Carlo simulations of the main components of *EuEO* in the presence of water molecules on the metal surface were also studied. The configurations of the studied compounds Uvaol (a), Betulinaldehyde (b), and d-Allose (c) reach an optimal equilibrium on the Fe (110)/100 H_2_O surface system, as shown in Figure 13. Furthermore, Table 8 shows the adsorption energy values obtained with respect to the MCS. According to the dynamic simulation results (Figure 13), the adsorption behavior of the principally studied essential oil compounds seems to be adsorption onto the mild steel surface [13]. This is evident from the negative adsorption energy values of these compounds on metal surfaces in the presence of water molecules (Table 8). Furthermore, the adsorption patterns of the molecules studied were identical. The physical and/or chemical bonds of a, b, and c are created through the donation of π electrons from the carbon nucleus and of electron pairs from oxygen atoms to the iron metal.

Moreover, Table 8 shows that the adsorption energies of the primary *EuEO* compounds are significantly greater than those of the H_2_O molecules, suggesting that the oil molecules may gradually replace the water and acid molecules on the Fe (110) surface [14]. As a result, the interaction of the various components of *EuEO* oil may create a more stable layer that can shield the steel surface from the harshness and attack of the hydrochloric acid environment [13], demonstrating the reliability and validity of the experimental findings.

## 3. Materials and Methods

### 3.1. Botanical Description of Eucalyptus

*Eucalyptus globulus*, a common plant in the Myrtaceae botanical family, is widely cultivated in Morocco. *Eucalyptus globulus* leaves do not change color from year to year. Reaching heights of 24 to 40 m, this tree has a sturdy trunk and smooth, tawny, or grayish-white bark. The *Eucalyptus globulus* leaves used in this study were naturally obtained in the fall (November) in the Ain Cheggag region, which is located 21 km outside of the Moroccan city of Fez.

### 3.2. Eucalyptus Leaf Powder Preparation

The eucalyptus leaves are harvested fresh, cleaned with water to wash away dirt, weeds, and small stones, and then rinsed with distilled water and allowed to air-dry for 48 h. The biomass is then dried for 48 h at 60 °C in an oven. Finally, an electric grinder is used to grind the dry biomass into an extremely fine powder (Eu). After that, the powder is sieved (using a 200 µm sieve) to collect particles smaller than 200 µm.

### 3.3. Characterization of Eu and EuEO

To characterize the properties of Eu and EuEO, a number of techniques were used:

- Using a Quanta 200 FEl-XRF device, environmental scanning electron microscopy (ESEM) in conjunction with an energy-dispersive X-ray (EDX) was utilized for microanalysis and morphology assessment. One of its main advantages is that it can evaluate the surface of wet or soft samples and scan materials that are delicate or hydrated. Moreover, a high-resolution picture of the powder’s surface features and microstructure is produced [64].
- Using KBr pellets, the FTIR spectra of the powdered eucalyptus leaves were displayed on a Nicolet Avatar 320 spectrophotometer. A total of 32 scans were recorded in transmittance mode between 4000 and 400 cm^−1^, with a resolution of 4 cm^−1^ [65].
- Thermogravimetric analysis (TGA) and differential thermal analysis (DTA) were performed using an SDT Q600 TA instrument in air, with heating from 30 °C to 1000 °C at a rate of 10 °C/min; alternatively, heating was carried out on Al_2_O_3_ crucibles using a Netzsch STA 449 F1 Jupiter thermal analyzer under a N_2_ atmosphere (100 mL/min) from 25 °C to 1000 °C at a rate of 10 °C/min. Data were processed via Netzsch Proteus 6.1 Thermal Analysis software [65].

### 3.4. Eucalyptus Leaf Essential Oil Extraction (EuEO)

Using a Clevenger hydrodistillation technique, we were able to obtain *EuEO*. It took about two hours to distill every sample. For every distillation, 200 g of powdered eucalyptus leaves was utilized. The average yield of essential oil was determined from the dry matter and reported as mL/100 g (*v*/*v*). Mass spectrometry (GC-MS) was used to examine the extracted essential oil (*EuEO*) [13,14].

### 3.5. GC/MS Analysis

For the purpose of the GC/MS analysis, a Hewlett-Packard apparatus was utilized, which was fitted with an apolar capillary column (RTxi-5 Sil MS) (30 m × 0.25 mm ID, 0.25 µm film thickness), with the temperature set from 50 to 250 °C at 5 °C/min, and connected to a mass spectrometer (HP 5973). Helium was utilized as the vector gas in fractionated mode, with a flow rate of 112 µL/min and a ratio of 1/74.7. By verifying their identities using MS (NIST98 spectra collection), the components were identified. A 1 μL sample was manually injected (1/50 in hexane) [13,14].

### 3.6. Mild Steel Preparation

Mild steel was the material of choice for this study because of its well-known conductivity and affordability across a wide range of industries. With less than 5% carbon, iron is its primary constituent. Only carbon significantly affects the characteristics of steel, though it can contain other elements as well. The chemical composition of mild steel is displayed in Table 9 [33].

### 3.7. Electrolytic Medium

The corrosive solution in this study is a molar hydrochloric acid solution made from a 37% hydrochloric acid solution with a specific gravity of 1.19 using distilled water, and the inhibitor concentrations made from 1 M HCl vary from 0.25 g/L to 1 g/L [33].

### 3.8. Electrochemical Method

A VersaSTAT 4 potentiostat controlled by EC-Lab V11.27 was used for the electrochemical measurements. This involved using a small glass cell with three electrodes. This cell, constructed from mild steel (1 cm^2^), had an Ag/AgCl electrode as the reference electrode and a platinum wire as the counter-electrode. Meanwhile, the rectangular test substrate, with a 1 cm^2^ exposed surface, was dipped into the sample for 30 min to establish an open-potential circuit [33].

(6) $$IE_{pdp}=\frac{i_{corr}-i_{corr_{inhi}}}{i_{corr}}\times 100$$

$i_{corr}$ and $i_{corr_{inhi}}$ represent the estimated corrosion current density with and without the inhibitor, respectively.

At the same time, potentiodynamic polarization was measured within the potential range of −600 to −200 mV Ag/AgCl at a 1 mV/s scan rate. For the EIS measurements of the OCP, 100 mHz to 100 kHz frequencies were obtained with an alternating current amplitude of ±10 mV.

These results are consistent with the two designs by Nyquist and Bode [33,66,67,68,69,70].

(7) $$IE_{IES}=\frac{R_{p_{inhi}}-R_{p}}{R_{p_{inhi}}}\times 100$$

$R_{p_{inhi}}$ and $R_{p}$, respectively, represent the charge transfer resistance without and with addition of the inhibitor.

### 3.9. Computational Study

The experimental behavior of this essential oil as a mild steel corrosion inhibitor in molar hydrochloric acid media was investigated. Moreover, the three main components contributing to the oil’s inhibitory performance were assessed using quantum chemical computations [71,72,73]. Density functional theory (DFT) was used to optimize the geometries of these compounds utilizing the B_3_LYP/6-31G (d, p) basis set and GAUSSIAN 09 software. To gain a better understanding of the inhibition mechanism, a number of quantum descriptors were calculated, including a molecular orbital (E_HOMO_ and E_LUMO_), the gap energy (ΔE), the dipole moment (μ), the electronegativity (χ), the hardness (η), the softness (σ), and the fraction of electron transferred (ΔN_110_) in the aqueous phase [74].

### 3.10. Monte Carlo Simulation

The inhibitor molecule adsorption behavior on the mild steel surface was studied using Monte Carlo simulation. An initial Fe (1 1 0) cell with a 50 Å edge was constructed and expanded into a supercell (10 × 10) in order to obtain the most stable adsorption model. An optimized simulation system was run using the Forcite module, incorporating 100 molecules of H_2_O by means of the COMPASS force field and employing the adsorption localization module in the Materials Studio software (2017) [74].

### 3.11. Surface Analysis

Mild steel samples, without and with the inhibitor at a 1 g/L optimum concentration, were analyzed in terms of their composition and surface morphology using the SEM-EDX spectroscopy method [33,70]. An environmental scanning electron microscope equipped with an EDX probe, model QUANTA 200, combined with energy-dispersive spectroscopy (EDX) at an accelerating voltage of 15 kV, was used for the analysis.

## 4. Conclusions

In this research, essential oil derived from *Eucalyptus globulus* (*EuEO*) leaf powder was evaluated as an effective green corrosion inhibitor for mild steel in a 1 M hydrochloric acid medium. A variety of techniques were used, involving intensity–potential measurements, electrochemical impedance spectroscopy (EIS), density functional theory (DFT) and Monte Carlo (MC) simulations. The results obtained prompt the following findings:

- The GC-MS analysis identified the major active chemicals in *Eucalyptus globulus* essential oil (EuEO): d-Allose (12.94%), Betulinaldehyde (31.75%), and Uvaol (32.17%).
- *EuEO* exhibited potent corrosion inhibition for mild steel in molar hydrochloric acid, achieving an inhibitory efficiency of up to 97% at a 1 g/L concentration.
- Significant adsorption of the *EuEO* molecules onto the mild steel surface was observed in the acidic medium.
- Polarization studies suggested that *EuEO* acts as a mixed-type inhibitor, blocking both cathodic and anodic reactions.
- Electrochemical impedance spectroscopy (EIS) indicated that *EuEO* significantly decreases the double-layer capacitance and increases charge transfer resistance.
- Surface analysis confirmed that *EuEO* forms a protective coating on the mild steel surface, adhering effectively.
- Monte Carlo (MC) dynamic simulations and density functional theory (DFT) simulations showed that *EuEO* functions as both an electron donor and acceptor to and from the iron surface.

This approach supports Morocco’s sustainable development agenda, emphasizing green chemistry by reducing and eliminating environmentally damaging substances.

## Figures and Tables

**Table 1:** Elements mass proportions as determined by EDX spectroscopy.

Element	O	C	K	Ca
**Eu**	46.5	49.66	1.63	2.21
**Eu+EtOH**	64.53	35.47	-	-

**Table 2:** Most abundant major constituents of *EuEO*.

	Name	Molecular Formula	Structure	Molar Mass (g/mol)
**a**	Uvaol	C_30_H_50_O_2_	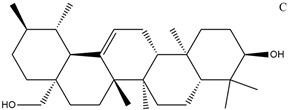	442.71
**b**	Betulinaldehyde	C_30_H_48_O_2_	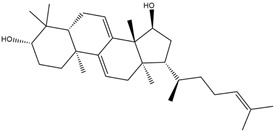	440.36
**c**	d-Allose	C_6_H_12_O_6_	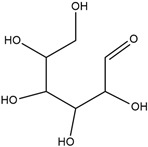	180.06

**Table 3:** Inhibitory efficiency and electrochemical parameters of different concentrations.

	Concentration (g/L)	−E_corr_ (mV/Ag/AgCl)	i_corr_ (µA.m^−2^)	−β_c_ (mV.dec^−1^)	−β_a_ (mV.dec^−1^)	IE_pdp_ (%)
**HCl 1 M**	-	451.506	793.261	123.8	84.5	-
***EuEO***	1.00	437.847	23.775	107.7	97.9	**97**
	0.75	422.626	35.334	103.4	76.8	**95**
	0.50	445.678	35.235	87.4	61.4	**95**
	0.25	441.017	92.280	88.9	64.2	**88**

**Table 4:** Impedance parameters of mild steel in a 1 M HCl solution with different concentrations of the inhibitor studied.

Medium	Concentration g/L	R_s_ (Ω.cm^2^)	R_p_ (Ω.cm^2^)	Q (µF.s^n−1^)	n_dl_	C_dl_ (µF.cm^−2^)	IE_IES_ %
**HCl 1 M**	-	1.22	33.97	315	0.82	117	-
***EuEO***	1.00	2.451	1113	42.46	0.799	19.69	**97**
	0.75	2.849	538.6	44.24	0.811	18.47	**94**
	0.50	3.154	431.5	56.37	0.795	21.63	**92**
	0.25	2.52	172.4	10.55	0.797	38.04	**80**

**Table 5:** Adsorption isotherm parameters of *EuEO* inhibitor obtained from EIS method at 298 K.

Isotherms	R^2^	Parameters		K_ads_ (L/g)	ΔG^0^_ads_ (kJ/mol)
**Langmuir**	0.999	Slope	1.002	34.36	−25.87
**Freundlich**	0.870	Z	0.065	0.95	−16.98
**Temkin**	0.874	a	−8.26	1.02 × 10^7^	−57.09
**El-Awady**	0.961	1/y	1.02	30.68	−25.59

**Table 6:** Elements mass proportions as determined by EDX spectroscopy.

Element	O	C	Fe	Ca	Cl	Si
**MS**	28.9	10	58	0.8	1.8	0.6
**MS + *EuEO***	25.3	2.3	72.5	-	-	-

**Table 7:** Quantum chemical descriptors for Uvaol, Betulinaldehyde, and d-Allose obtained in aqueous phase.

Parameters	Uvaol	Betulinaldehyde	d-Allose
**E_HOMO_ (eV)**	−6.0949	−6.6873	−7.1510
**E_LUMO_ (eV)**	−0.8305	−0.9293	−0.9911
**ΔEgap (eV)**	5.2644	7.6166	8.1421
**η (eV)**	2.6322	3.8083	4.0711
**σ (eV^−1^)**	0.3799	0.2626	0.2456
**χ (eV)**	3.4627	2.8790	3.0800
**ΔN_110_**	0.2578	0.2548	0.2137
**μ (D)**	3.6619	4.5603	4.4922

**Table 8:** MC parameters for studied major constituents of *EuEO* on Fe (110).

Systems	Total Energy (kcal/mol)	E_ads_ (Inhibitor) (kcal/mol)	E_H2O_ (Inhibitor) (kcal/mol)
**Fe(110)/a/100H_2_O**	−2803.122	−4980.527	−35.61
**Fe(110)/b/100H_2_O**	−2833.019	−5015.946	−33.80
**Fe(110)/c/100H_2_O**	−2658.588	−4944.691	−33.13

**Table 9:** Chemical composition of steel components used.

Elements	Fe	Si	C	Mn	S	P	Al
**(%) Mass**	99.21	0.38	0.21	0.05	0.05	0.09	0.01

## Data Availability

The original contributions presented in the study are included in the article.
